# Supplementary material for: Adult Ossabaw Pigs Prefer Fermented Sorghum Tea over Isocaloric Sweetened Water
Source: Animals (Basel). 2023 Oct 18;13(20):3253. doi: 10.3390/ani13203253 (PMC10603632; doi:10.3390/ani13203253)
Supplement: Supplementary file 1 [file animals-13-03253-s001.zip › S2TransposeCode.pdf]

```
%let path=C:\Users\huiwu\OneDrive - Kansas State University\KSU\others\Lindsey
Hulbert\Data;
PROC IMPORT OUT= WORK.swine
DATAFILE= "&path/2022_0718_Preference_LEH.xlsx"
DBMS=EXCELCS REPLACE;
SHEET="SAS Ready";
SCANTEXT=YES;
USEDATE=No;
SCANTIME=YES;
RUN;
*options validvarname=any;
/*FILENAME REFFILE
'/home/wuhui570/sasuser.v94/others/Other/2022_0718_Preference_LEH.xlsx';
PROC IMPORT DATAFILE=REFFILE
DBMS=XLSX
OUT=WORK.swine;
SHEET="SAS Ready";
GETNAMES=YES;
RUN;*/
*data refusal;
data refusal;set swine;
where phase_='Test_4_5_6';
if gender_ ='gilt' then gender_ ='Gilt';
keep pig_id Tech_ phase_ gender_ Pen left_pod Mid_pod Right_pod rep
left_refusal_ml Mid_refusal_ml Right_refusal_ml;
run;
data long;set refusal;
pos='Right_Pod';
trt=right_pod;
output;
pos='Left_Pod';
trt=left_pod;
output;
pos='Mid_Pod';
trt=mid_pod;
output;
drop left_pod mid_pod right_pod;
run;
data long; set long;
if pos='Left_Pod' then do; resp=left_refusal_ml;end;
else if pos='Mid_Pod' then do; resp=mid_refusal_ml;end;
else if pos='Right_Pod' then do; resp=right_refusal_ml;end;
run;
/*
data long; set long;
resp=input(resp1, 6.2);
drop resp1;
run;*/
*summary statistic;
OPTIONS ORIENTATION=PORTRAIT NODATE;
ods rtf file="&path\SASoutput\output_Refusal_8_18_2022.doc"
STYLE=CHROMEPRINTER;
*SUMMARY STATISTICS;
TITLE 'SUMMARY STATISTICS for Refusal';
TITLE2 'Data listing';
proc tabulate data=long STYLE=[JUST=C];* out=result;
format pig_ID 6.0 rep 6.0;
CLASS pig_ID rep trt;
```
